# Restricted regions of enhanced growth of Antarctic krill in the circumpolar Southern Ocean

**DOI:** 10.1038/s41598-017-07205-9

**Published:** 2017-07-31

**Authors:** Eugene J. Murphy, Sally E. Thorpe, Geraint A. Tarling, Jonathan L. Watkins, Sophie Fielding, Philip Underwood

**Affiliations:** 1 British Antarctic Survey, NERC, Cambridge, UK; 2 UNEP World Conservation Monitoring Centre, Cambridge, UK

## Abstract

Food webs in high-latitude oceans are dominated by relatively few species. Future ocean and sea-ice changes affecting the distribution of such species will impact the structure and functioning of whole ecosystems. Antarctic krill (*Euphausia superba*) is a key species in Southern Ocean food webs, but there is little understanding of the factors influencing its success throughout much of the ocean. The capacity of a habitat to maintain growth will be crucial and here we use an empirical relationship of growth rate to assess seasonal spatial variability. Over much of the ocean, potential for growth is limited, with three restricted oceanic regions where seasonal conditions permit high growth rates, and only a few areas around the Scotia Sea and Antarctic Peninsula suitable for growth of the largest krill (>60 mm). Our study demonstrates that projections of impacts of future change need to account for spatial and seasonal variability of key ecological processes within ocean ecosystems.

## Introduction

Energy flow to the highest trophic levels in polar ocean ecosystems is dominated by a small number of species, which are highly abundant and have life-cycles that reflect the strong seasonality of these high latitude regions^1–3^. In the Arctic, food webs are more heterogeneous than in the Antarctic because of the influence of lower latitude species, but a few species of zooplankton and fish are the major prey of most of the larger predators in higher Arctic regions^3–6^. In some areas of the Southern Ocean, ice krill (*Euphausia crystallorophias*), silverfish (*Pleuragramma antarcticum*) and mesopelagic fish species are important^7–9^. However, the single most important prey species in Southern Ocean food webs is Antarctic krill (*Euphausia superba* Dana; hereafter referred to as krill). It is the main agent of energy transfer in a short two-step food chain from primary production, maintaining globally important populations of seabirds and marine mammals^7, 10^. The short, krill-characterised, food chain dominates the pathways of energy flow to higher trophic levels during the summer months over large areas of the Southern Ocean^7–12^. The food webs are, however, complex, krill are omnivorous, and other pathways of energy flow are important in maintaining the overall structure, functioning and resilience of these ecosystems^3^. These pathways include microbial components and smaller zooplankton, which also provide alternative food sources for krill as they grow and develop, and in regions or periods (e.g. winter) when new production is limited^3^.

In coastal regions associated with the northern Antarctic Peninsula, the South Orkney Islands and around South Georgia, krill are also the target of a commercial fishery, in which catch rates have increased in recent years^13^. In the same regions over the last 50 years upper ocean temperatures and sea ice concentration have shown marked changes, which are related to variation in atmospheric processes^14^. Over the same period, there have also been changes in regional ecosystem structure and functioning but the mechanisms involved are poorly understood^7, 15–18^.

Krill has a life-cycle that is closely associated with the winter sea-ice and a circumpolar distribution that is influenced by the oceanic circulation, which is dominated by the Antarctic Circumpolar Current (ACC)^7, 12, 18, 19^. Potential future warming and reductions in sea-ice and ocean pH could reduce krill reproductive output, recruitment success and growth in these regions^20–26^. Such perturbations are expected to change the distribution and abundance of krill and impact overall food web structure and functioning^8, 27^.

To generate projections of the impacts of such physical and chemical changes on krill distribution, and the wider ecosystem, requires an understanding of how variation in population processes affects circumpolar krill distribution. Although there is detailed information available on aspects of the krill life-cycle in some regions, there is little quantitative information on the distribution, abundance or basic biology of krill throughout much of the Southern Ocean^28–33^. Understanding of the processes affecting larval krill development and dispersal has increased over the last decade^34–36^. Information is also available on what determines the growth and development of adult krill in different regions, but there remain major gaps in understanding^32, 33, 37, 38^.

The prevailing view of the distribution of krill is that it is circumpolar but asymmetric, with a large proportion of the population (>50%) occurring in the Atlantic sector of the Southern Ocean^12, 39^. Elsewhere around the Antarctic the distribution is less well characterised, but the general pattern, based on recent quantitative analyses, suggests a generally continuous distribution, with some variations in relative abundance and northern latitudinal extent^12, 32, 40–44^. One possible measure of the suitability of habitat is the growth potential, which should be reflected in the size structure of krill populations observed around the Southern Ocean. Krill are known to both grow and shrink over the course of their life-cycle^45^ which is believed to be the result of both the availability of food resource^46^ and the seasonal pattern of reproduction^45^. Although the size structure of krill is known to vary in particular regions of the Southern Ocean^47^, there has been little systematic assessment of circumpolar variation in size structure of populations.

We examine the validity of the view that regional variation in krill growth can lead to asymmetries in its distribution and size structure. We use satellite-derived estimates of mean monthly sea surface temperature (SST) and chlorophyll-*a* concentration data to provide circumpolar fields with a seasonal resolution. We use these data to estimate growth of different sized krill throughout the Southern Ocean for every month and location where data are available utilising an existing growth rate model^10, 37^. The model was derived from estimates of instantaneous growth rate across a range of environmental conditions^37^. Here we use the model to estimate potential growth based on the seasonally changing environmental conditions. We develop a series of alternative calculations to examine the sensitivity of the results and specific regional analyses are undertaken to examine in detail the changes in krill size through the year.

## Results

### Seasonal development of krill growth

**Figure 1:**
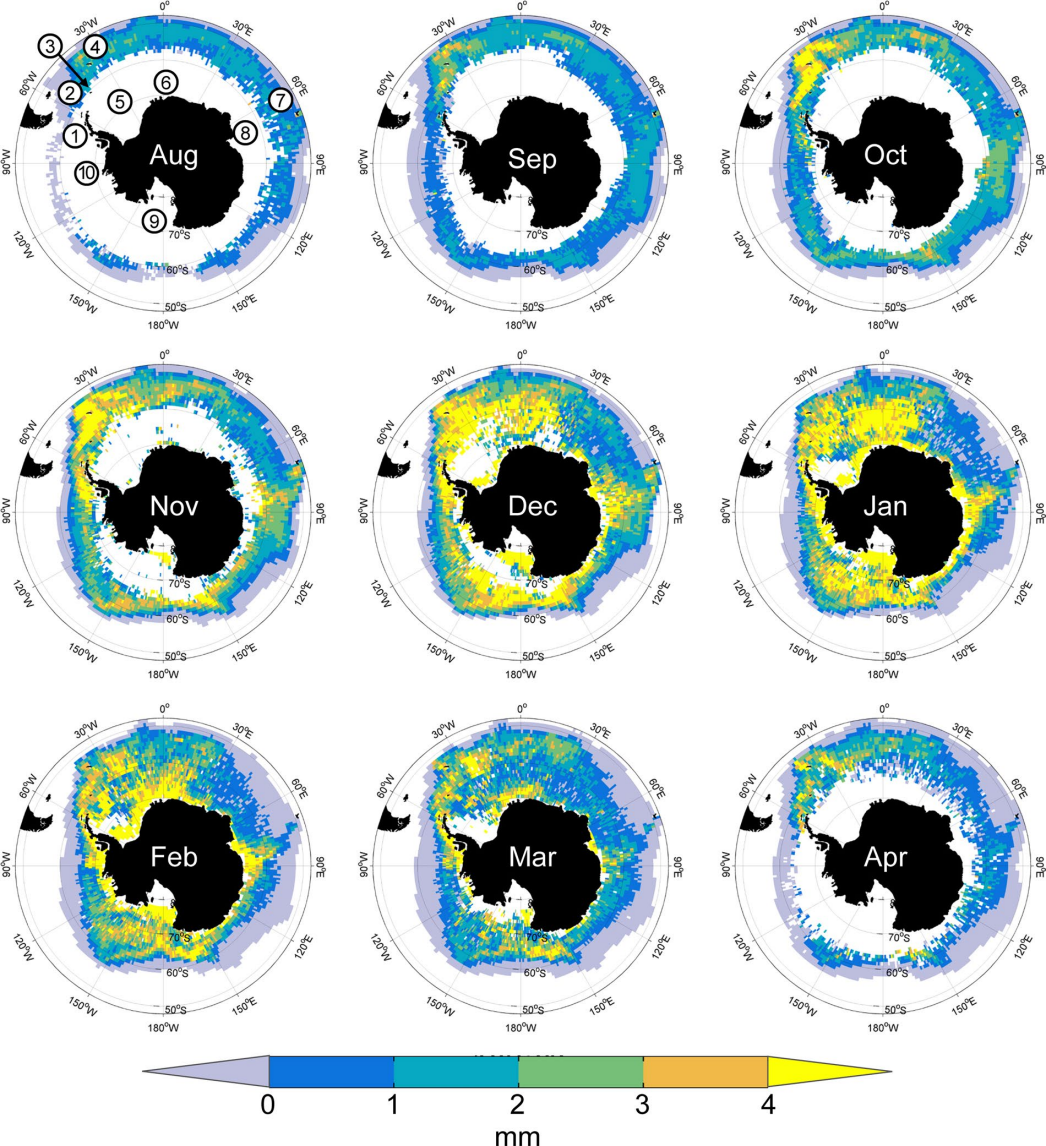
Mean monthly potential growth (daily growth rate x number of days in month) for 40 mm krill calculated for grid cells where both temperature and chlorophyll *a* data are available. White shading indicates missing environmental fields or temperature >5 °C. Panel 1 labels: 1 – West Antarctic Peninsula, 2 – Scotia Sea, 3 – South Orkney Islands, 4 – South Georgia, 5 – Weddell Sea, 6 – Lazarev Sea, 7 – Kerguelen Plateau, 8 – Prydz Bay, 9 – Ross Sea, 10 – Amundsen Sea. Figure created with Matlab software version R2013a (www.mathworks.com) using M_Map mapping toolbox version 1.4f (https://www.eoas.ubc.ca/~rich/map.html).

There are marked regional differences in the seasonal development of growth conditions around the Southern Ocean. At the beginning of spring (September) the potential growth of 40 mm krill (taken as a mid-size reference value, krill reach a maximum body length of about 65 mm) is greatest in the Scotia Sea region and in the northern areas around South Georgia (Fig. 1). This region of higher positive potential growth rates (>3 mm month^−1^) develops southward during October and November. By December it extends across the Scotia Sea encompassing ice-free regions in the south and around the Antarctic Peninsula in the west. By January, the main centre of high potential growth has shifted south and east towards the eastern Weddell Sea and Lazarev Sea region between ~ 20°W and 20°E and 60°S to 65°S and close to the continent. Potential growth rates begin to reduce (<3 mm month^−1^) in many areas during February and March but continue to be positive across the Scotia Sea region into early autumn, and northern regions could still potentially support positive growth rates until April (Fig. 1). These shifting regions of high potential growth reflect the changing surface temperatures and chlorophyll *a* levels. For example, during January in areas north of South Georgia, mean chlorophyll *a* concentrations are ~1 mg chl *a* m^−3^, but the high SSTs (>3 °C) limit potential growth rates (Supplementary information, Figure S3). In contrast, over much of the Lazarev Sea at this time chlorophyll *a* concentrations are >0.5 mg chl *a* m^−3^ and SSTs are between 0.5 °C and 1 °C resulting in generally high potential growth rates (>3 mm month^−1^).

There are two further regions where the model indicates that potential growth rates for 40 mm krill are high (>3 mm month^−1^) by December and these develop further southward during January through to March. The first is centred between the north of Prydz Bay and the southern Kerguelen Plateau between ~ 80°E and 110°E and 60°S and 50°S, developing towards the Antarctic continent coast by January (Fig. 1). The second area occurs to the north of the Ross Sea between ~150°E and 150°W and 70°S and 60°S (Fig. 1). Through January and February areas further south and around much of the coast become high potential growth habitats. Between May and July sea ice dominates southern regions and there are very few data available on chlorophyll *a* concentrations due to cloud and sea ice cover. By August, some data are again available and show that the more northern regions can support positive potential growth even at this time of year. These 3 areas of strong positive potential growth contrast strongly with the 3 in-between regions of generally low (<2 mm month^−1^) or negative potential growth rates (shrinkage) through most of the year (Fig. 1). These are areas in the Amundsen Sea (~120°W to 80°W), Indian Ocean (~30°E to 60°E) and South Pacific regions (~120°E to 150°E).

**Figure 2:**
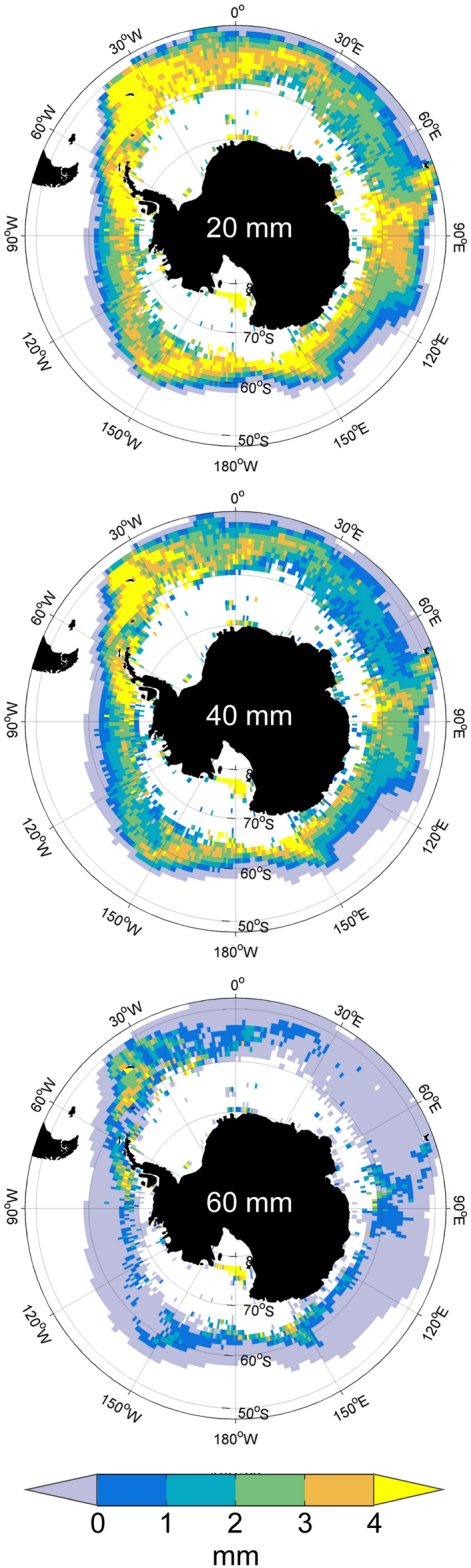
Mean monthly potential growth (daily growth rate x number of days in month) of krill for November calculated for grid cells where both temperature and chlorophyll *a* data are available. Calculated for 20 mm (top), 40 mm (middle) and 60 mm (bottom) krill (see also Fig. 1). Figure created with Matlab software version R2013a (www.mathworks.com) using M_Map mapping toolbox version 1.4f (https://www.eoas.ubc.ca/~rich/map.html).

In late spring (November) potential growth rates of 20 mm krill are, as expected^37^, higher throughout the Southern Ocean than those of the 40 mm krill, but the circumpolar pattern is very similar and with the highest rates (>3 mm month^−1^) centred on the same three regions (Fig. 2). The areas of positive potential growth of 60 mm krill are much more restricted, with a few areas of enhanced potential growth (>2 mm month^−1^) mainly in the Scotia Sea and around the Antarctic Peninsula (Fig. 2).

**Figure 3:**
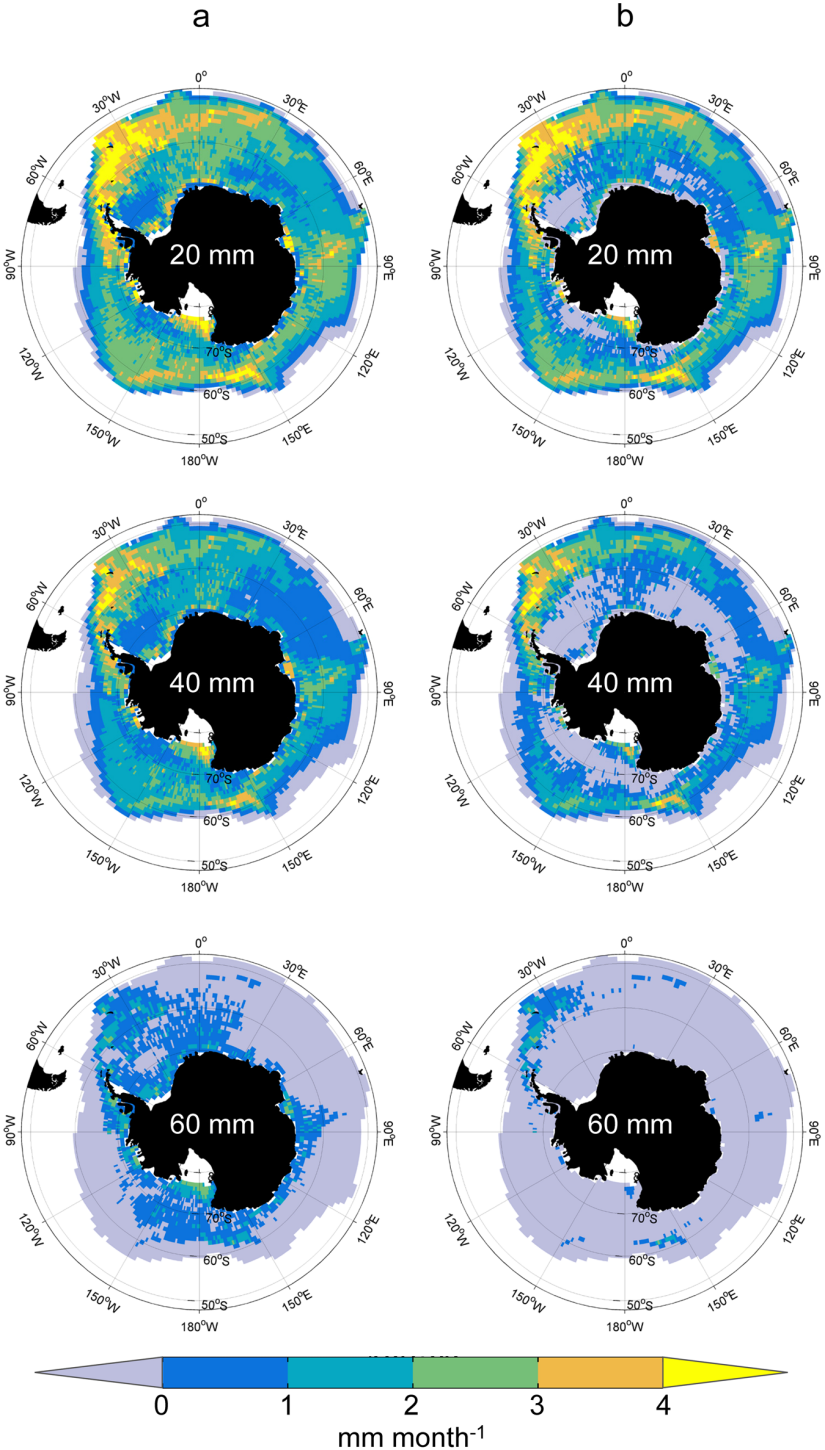
Mean monthly potential growth rate for increasing krill size calculated from September to April (mm month^−1^) for two different assumptions about missing chlorophyll *a* data. Panel **a** plots based on assumption that missing chlorophyll *a* data are periods of 0 mm month^−1^ growth. Panel **b** plots based on assumption that missing chlorophyll *a* data signifies a value of 0 mg chl *a* m^−3^. Only cells where each monthly temperature <5 °C are used (see also Fig. 1). Figure created with Matlab software version R2013a (www.mathworks.com) using M_Map mapping toolbox version 1.4f (https://www.eoas.ubc.ca/~rich/map.html).

The circumpolar pattern of mean monthly potential growth rate (August to April) for 20 mm and 40 mm krill shows the general pattern of 3 regions of higher potential growth rates separated by 3 regions of generally lower rates (Fig. 3a). Assuming that the periods of no chlorophyll *a* data correspond to no available food (Fig. 3b), rather than no growth, results in more areas of negative potential growth rates in southern regions and reduces the mean potential growth rates in these areas (cf. Fig. 3a and b). Mean monthly potential growth rates are negative in areas of more persistent ice cover in the Weddell and Ross Seas and in the warmer regions around the Polar Front. For the largest 60 mm animals we do not expect to see high potential growth rates^11, 37^ and the areas of positive mean monthly potential growth rates are much more restricted than for smaller krill (Fig. 3). Mean positive potential growth rates for large krill are possible in the 3 main centres of potential growth and in areas close to the coast (Fig. 3a); the areas of enhanced potential growth (>1 mm month^−1^) are further limited mainly to areas across the Scotia Sea and around the northern Antarctic Peninsula and South Georgia when it is assumed that no food is available when chlorophyll *a* data are missing (Fig. 3b). There is also marked seasonality in potential growth rates and the areas over which positive potential growth occurs for the different sized krill (Table S1). For both 20 mm and 40 mm krill positive potential growth of krill occurs over an area equivalent to 79% of the summer area during spring and over 50% during autumn (Table S1) and even for 40 mm krill potential growth rates >2 mm month^−1^ are maintained over ~25% of the area during autumn. For 60 mm krill, positive potential growth is possible during summer over ~50% of the area where 40 mm krill grow positively, but is more restricted during the rest of the year (Table S1).

### Regional variation in growth

**Figure 4:**
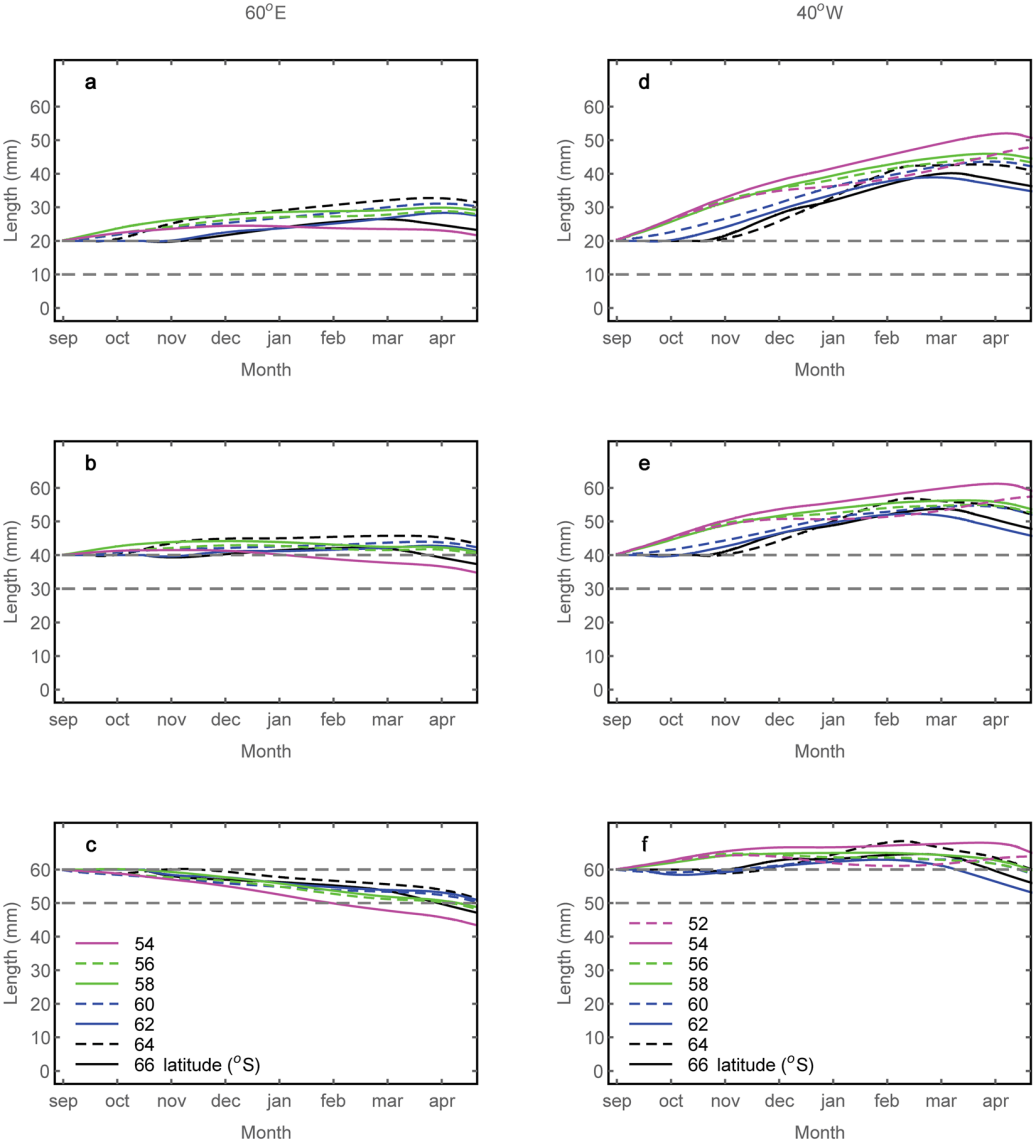
Growth curves at different latitudes along two longitudes from September to mid-April: 60°E (Indian Ocean sector, left panels (**a–c**)) and 40°W (Scotia/Weddell Seas, right panels, (**d–f**). Initial sizes of 20 mm (**a**,**d**), 40 mm (**b**,**e**) and 60 mm (**c**,**f**) and dashed lines show initial size and initial size minus 10 mm. Colours indicate latitude. Figure created using Wolfram Mathematica version 11 (https://www.wolfram.com/mathematica).

To assess the possible impacts of seasonal and spatial variation in potential growth (Fig. 1) we examined the expected change in krill size from spring to autumn (September–April) in two contrasting regions of weak and strong potential growth (respectively 60°E and 40°W; Fig. 4). In the region where potential growth is limited (60°E) a net positive increase between spring and autumn is possible for 20 mm krill and at some latitudes for 40 mm animals, with a later start to the growing season further south. There is no simple latitudinal relationship with potential growth rate and instead it is the mid-latitudes (~56 to 60°S) where the potential growth rates are highest (Fig. 4a and b). Larger krill (60 mm; Fig. 4c) are unable to maintain positive growth rates in this area, and there are only a few months, at mid-latitudes, where positive potential growth rates occur. The growth curves in the high potential growth Scotia-Weddell Sea region (40°W; Fig. 4d,e) show a general latitudinal gradient of higher potential growth in the north (~52 to 56°S) and lower potential growth in the south (~62 to 66°S), but they cross over indicating that the optimal growing conditions are found in different regions through the year. There is consistent positive potential growth over much of the Scotia Sea (areas north of 60°S) from about September until March or April for 20 mm and 40 mm krill. In southern areas all the krill can grow rapidly later in the season (January-March) and higher potential growth rates could be maintained by movement further north during the early winter. This further highlights that the location where optimal potential growth rates can be achieved changes over time. There is positive potential growth of the largest (60 mm; Fig. 4f) krill in the spring (September-November) in the more northern regions while, in areas further south in the Scotia Sea, positive potential growth occurs later in the season as expected. Only in the most northern regions in the high potential growth region (40°W) can 60 mm krill maintain their size between spring and autumn.

**Figure 5:**
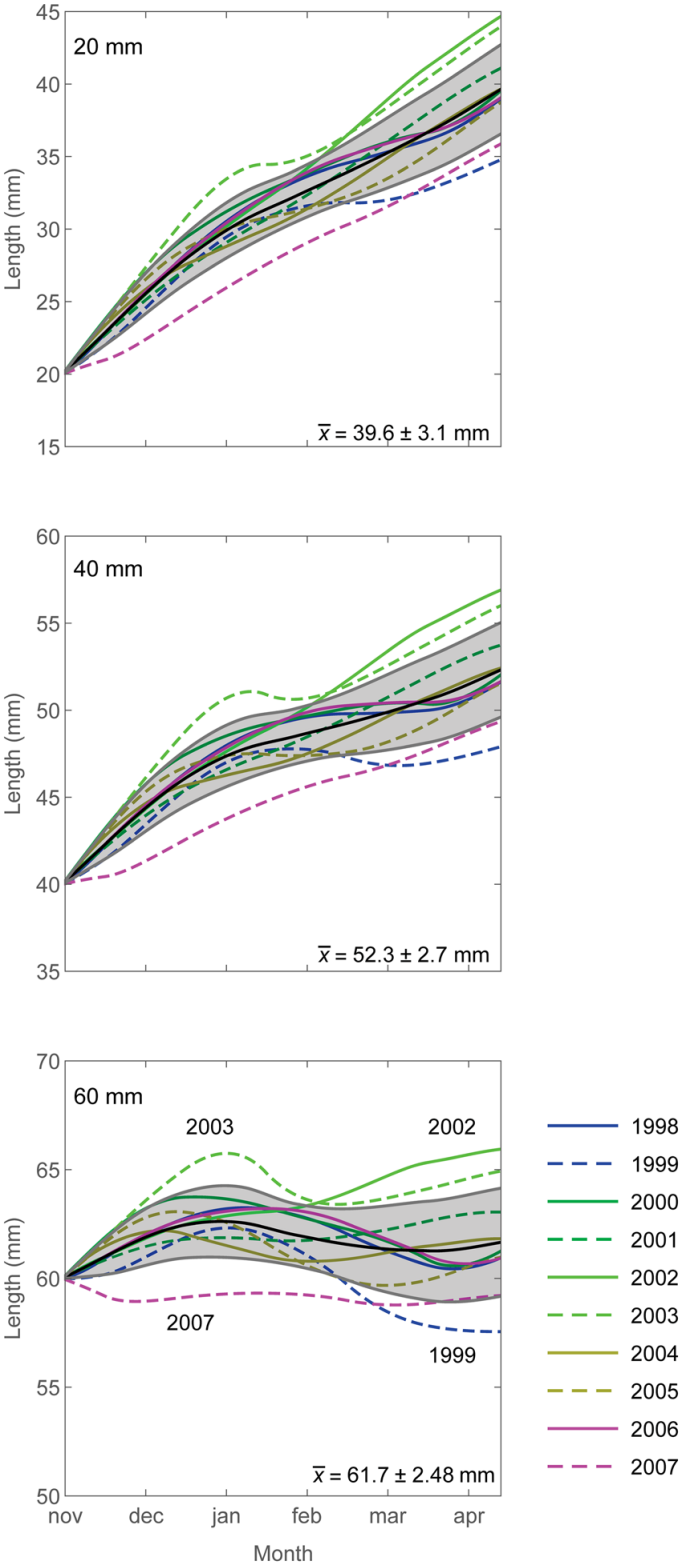
Growth curves (November to mid-April) from 1998 to 2007 at 54°S, 40°W for krill with initial sizes of 20 mm, 40 mm and 60 mm (1998 = austral summer 1997/1998). The colours show the growth curves for different years (see lower panel for key and annotation) along with the mean (black) and standard deviation (grey shading). Mean +/−1 standard deviation of the krill length by ~mid April (13^th^ April) are shown. Figure created using Wolfram Mathematica version 11 (https://www.wolfram.com/mathematica).

We examined the effects of interannual variability in sea surface temperature and food availability (chlorophyll *a*) (Figure S1) on growth rates in the high potential growth region of the northern Scotia Sea (40°W, 54°S). There is marked variation in potential growth rates, and hence change in size, between years (Fig. 5), resulting in a large range of krill sizes by late summer/autumn (~13^th^ April; Fig. 5). The difference is most marked for the very largest krill (60 mm), with net positive summer potential growth during some years (e.g. 2001/2002 and 2002/2003), while conditions in other years (e.g. 1998/1999 and 2006/2007) result in a reduction in size (Fig. 5). To generate a quantitative perspective of the impact of such interannual variation in the mean length on regional biomass, we examined the potential importance of variation in growth on estimated biomass of krill at South Georgia^48^ (Table S2). The analyses indicate that growth variation between years could generate substantial differences in biomass (Table S2). The range between low and high potential growth rate (resulting in ±4 mm difference in total length in December-January) estimates of biomass across the years is between 1.6 gm^−2^ in 2000 and 80.7 gm^−2^ in 2002. Such growth variation could be a factor in the observed interannual pattern of biomass variability and in determining the availability of krill to predators^48, 49^. For example, taking the lower quartile value in the observed series as an indication of a low biomass year (26.7 g m^−2^), there are 4 years in the observed series below this level, with 6 predicted in the low growth and 3 in the high growth series.

### Controls on circumpolar distribution and abundance

**Figure 6:**
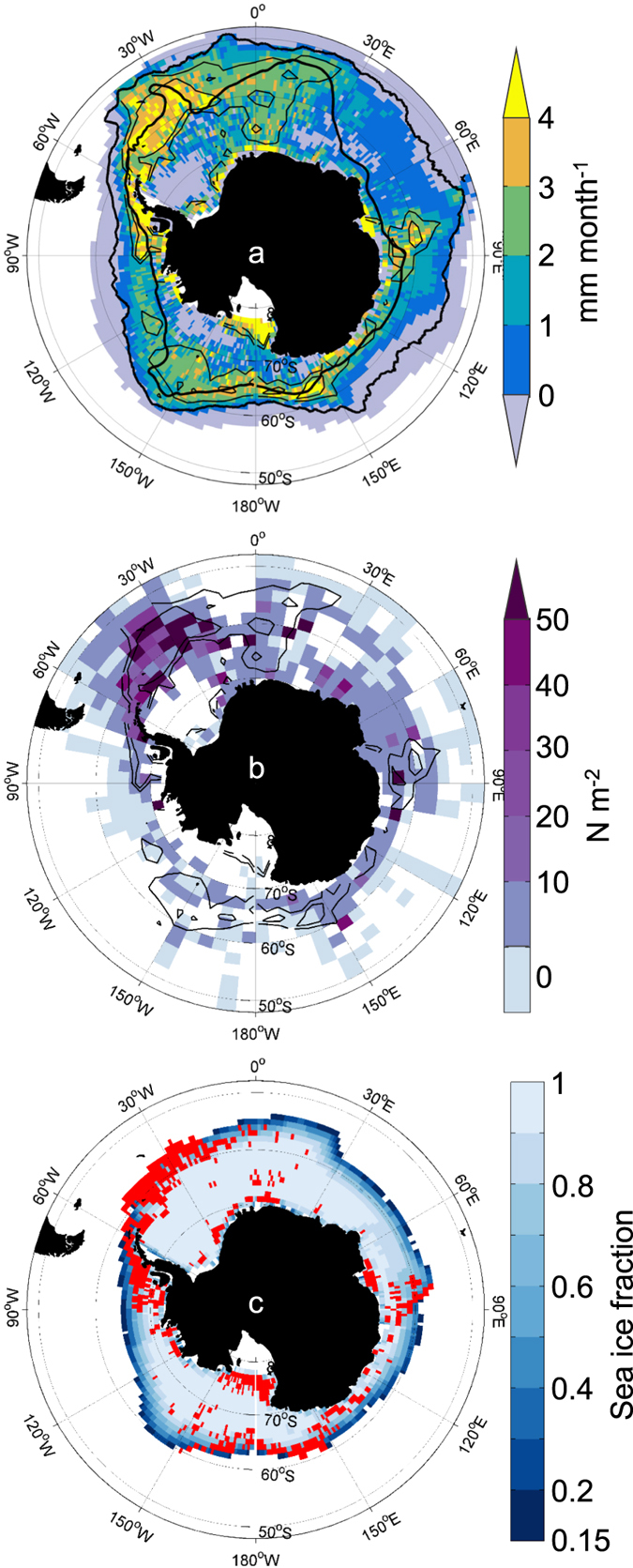
Circumpolar distributions: (**a**) Mean monthly potential growth rate of 40 mm krill for November to March (mm month^−1^); cells with missing data for chl *a* set to 0 mg m^−3^. White shading indicates cells with missing environmental data or temperature >5 °C. Contours of mean growth rate of 2 mm month^−1^ and 3 mm month^−1^ are derived from a smoothed version of the growth matrix (5° lon × 2° lat; thin black lines). Superimposed with mean positions of Antarctic Circumpolar Current (ACC) fronts^70–72^: Polar Front and southern boundary of the ACC (thick black lines). (**b**) Abundance of krill (N m^−2^) from KRILLBASE^69^ overlaid with contours of mean growth rate of 2 mm month^−1^ and 3 mm month^−1^ from Fig. 6a (thin black lines). (**c**) Regions where mean monthly potential growth rate of 40 mm krill over November to March exceeding 3 mm month^−1^ (from Fig. 6a) coincides with sea ice fraction in range 0.15 to 0.75 in any month (red). Background shading shows climatological mean sea ice fraction for October. Figure created with Matlab software version R2013a (www.mathworks.com) using M_Map mapping toolbox version 1.4f (https://www.eoas.ubc.ca/~rich/map.html).

To examine whether growth rate is important in determining the distribution and abundance of krill we compared the model potential growth rates with net-based abundance data^17^ (Fig. 6). The regions of highest recorded abundance of krill coincide reasonably well with the predicted areas of highest potential growth rates of 40 mm krill between November and March (Fig. 6a,b), occurring along the west Antarctic Peninsula, in the Scotia Sea, Lazarev Sea and Prydz Bay area, and dispersed around the continent close to the shelf. The exception is the offshore Ross Sea region (Fig. 6b), where the data do not show high krill abundance, but the model indicates that potential growth rates are high. This discrepancy suggests that additional population processes may be important in this region. However, the sampling intensity on which the abundance map is based is also highly variable and there are generally fewer samples from the offshore region of the Ross Sea.

Survival of krill larvae during winter is thought to be mainly dependent on their overwintering in marginal sea-ice environments. Locations where high adult potential growth rates during summer coincide with marginal ice zones during winter might be expected to be areas where the life cycle of krill (adult growth, survival, development to spawning and larval survival) is most likely to be completed successfully. We identified locations where the mean potential growth rate from November to March was >3 mm month^−1^ and sea ice concentration was between 15% and 75% in at least one month of the year (Fig. 6c). These locations are in the southern areas within the three main regions of high potential growth, especially in the Scotia Sea – Antarctic Peninsula region and intermittent areas nearer the coast (Fig. 6c).

**Figure 7:**
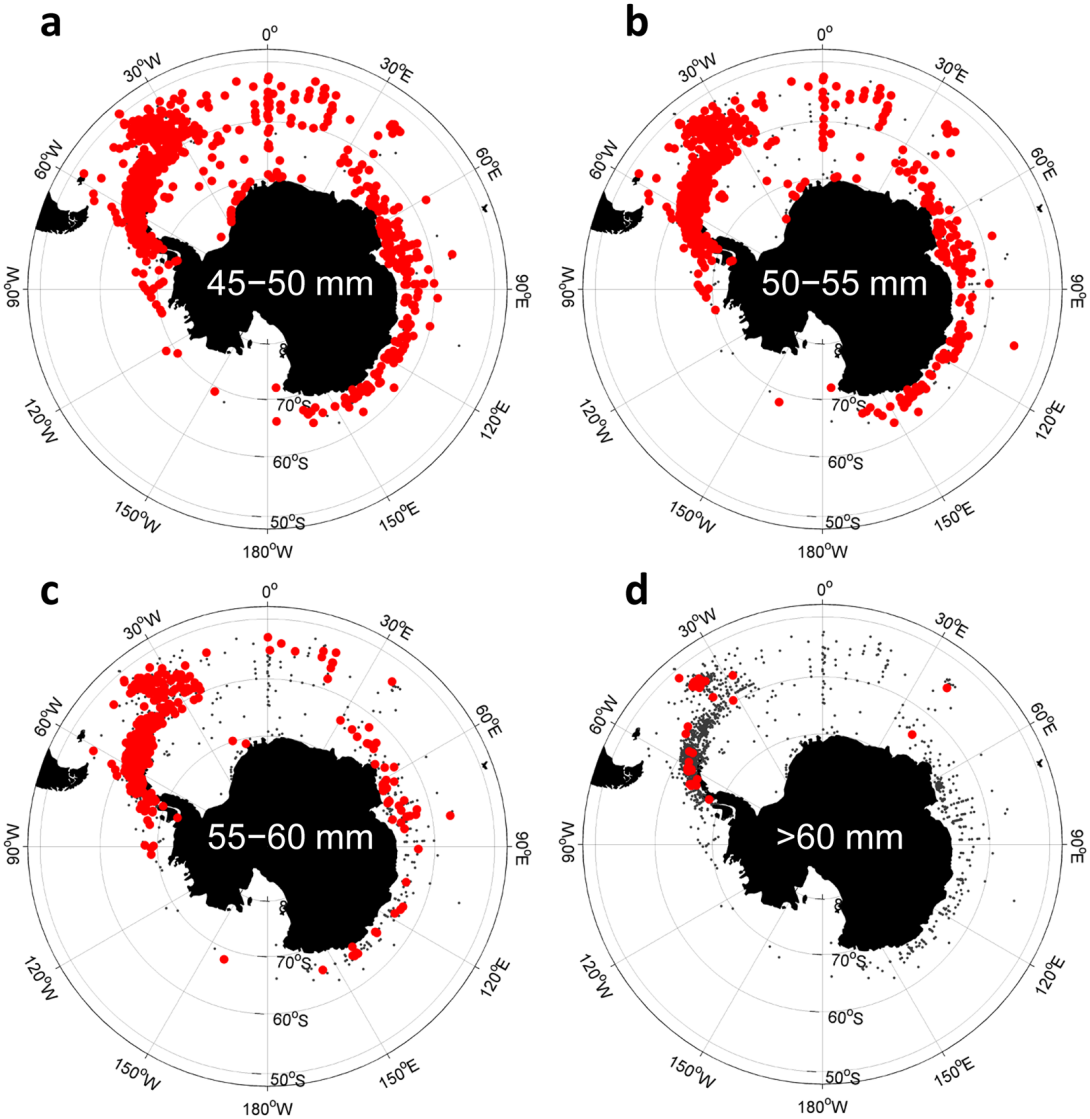
Distribution of krill in different size ranges recorded in net samples: (**a**) 45 to 50 mm, (**b**) 50 to 55 mm, (**c**) 55 to 60 mm and (**d**) >60 mm. Red dots show presence, grey dots absence of each krill size from net samples. Data from KRLLBASE^69^. Figure created with Matlab software version R2013a (www.mathworks.com) using M_Map mapping toolbox version 1.4f (https://www.eoas.ubc.ca/~rich/map.html).

The differences in potential growth rates with size that the model generates are marked (Figure S3 ^37^). While much of the Southern Ocean is suitable for positive growth of smaller sub-adult krill there are very few areas where positive growth rates are possible for the largest krill. These can only be maintained for a few months in areas of the highest phytoplankton concentration. To test this prediction we plotted the locations of larger krill (45 mm to >60 mm) obtained in circumpolar net samples (Fig. 7). The model predictions are generally consistent with the available data. Krill between 45 and 50 mm in size are more widely distributed than the largest krill >60 mm, which are almost completely restricted to the areas around the shelf in the Scotia Sea region, around South Georgia and the west Antarctic Peninsula (Fig. 7).

## Discussion

Our study indicates that a major factor in determining the distribution and abundance of post-larval krill is the marked geographical variation in seasonal growth. Our findings show that the circumpolar pattern of variation of krill potential growth is dominated by both the regional differences in the estimated summer mean concentration of chlorophyll *a* ^50^, and the duration of conditions that are favourable to phytoplankton growth. It is, therefore, the more northern regions in which chlorophyll *a* concentrations are high for extended periods and where annual primary production is also high^50^ where seasonal krill growth potential is most extensive^47^.

In contrast to a recent abundance-based empirical study that suggested a generally continuous circumpolar distribution of favourable habitat over much of the Southern Ocean^40^, our results indicate that variation in food availability results in three main regions in the ACC where conditions for growth are optimal, separated by three regions of lower growth. These are areas of high primary production where blooms occur regularly, chlorophyll *a* concentrations are generally greater than 0.5 mg m^−3^ and phytoplankton communities tend to be dominated by diatoms^51^. Krill can feed on a variety of prey items including different groups of diatoms, ice algae, dinoflagellates, copepods and benthic algae and detritus associated with the sediments and sea bed^29, 47, 52^. However, during the main summer growth period, in areas of high krill abundance, the diet of post-larval krill is dominated by diatoms, which are important in development of their reproductive capacity and for generating overwintering energy stores^47, 53^. The regions of high potential growth rate will, therefore, be expected to be the areas of high spawning potential.

The extent to which these areas of high potential growth are also population centres will, however, depend on a range of factors which can affect different stages of the krill life cycle^32, 34, 54–57^. The results show that a high growth potential occurs in areas where krill are abundant around the Antarctic Peninsula, Scotia Sea, Kerguelen Plateau and around the edges of the continent. However, the results also indicate that high potential growth alone is not sufficient to maintain a large population and that other factors are important. This is demonstrated in the region north of the Ross Sea where potential growth is high, but evidence on the importance of this area in terms of krill abundance is equivocal^32^. For large populations of krill to be maintained a combination of factors is likely to be important, including high potential growth, shelf areas where retention can occur, adjacent deep waters where successful spawning and egg development can be completed and within the region of seasonal advance and retreat of sea ice which is important in retention and larval survival.

Our results indicate that the high potential growth rates possible in northern regions, which may begin very early in the year (September and October), may result in krill being much larger much earlier than expected from studies undertaken further south, which may confound our understanding of the recruitment potential of krill in northern regions^58^. There is also a strong seasonal pattern of development of optimal conditions from northern regions to the south as summer progresses and then northwards again as autumn conditions develop. Through interactions with the ocean currents and drifting sea-ice there may be routes of connection that link the seasonally changing regions allowing krill to experience optimal growth conditions.

The model generates potential growth rates consistent with independent estimates derived using a range of methods applied throughout the Southern Ocean^11, 33, 37, 38^. In considering the limitations of the model the parameterisation indicates that the exact outcomes are sensitive to changes in parameter values (Tables S3 and S4; Figure S3), but the general circumpolar pattern of growth potential is robust. The model is based on the derived relation with SST and SeaWiFS estimates of chlorophyll *a* concentration^37^. This relationship may vary regionally and alternative satellite-derived estimates which generate a different chlorophyll distribution will affect the exact estimates and patterns of growth potential^59, 60^. In addition, changing productivity associated with day length may affect daily rates of krill growth. Satellite estimates cannot represent alternative food sources, including sub-surface chlorophyll, heterotrophic organisms and different phytoplankton community compositions, all of which may be important. We have not applied the model to the winter period, because the original study did not include data for the winter months, although data were included for southern and recently ice-dominated regions where chlorophyll *a* concentration was low and the krill underwent shrinkage^37^. The model-estimated shrinkage rates during periods of no or low food availability in favourable areas for growth in the Scotia Sea during summer (Fig. 4) are consistent with the limited information available (~−5 mm over winter for female krill)^45^. Krill have the capacity both to shrink and grow at each moult depending on the consumed level of resource during the preceding moult cycle^61^. The length of the moult cycle itself is not determined by energetic balance but is an internal physiological cycle that is sensitive to temperature and age^62^.

Shrinkage may be avoided through access to alternative food sources^47, 52, 63, 64^ or the reduction of metabolic rates and maintenance costs^34, 65^. To maintain positive growth rates for extended periods, food concentrations would have to be equivalent to >0.5 mg chl *a* m^−3^, which is unlikely in most regions of the deep ocean in ice-covered regions during winter. Developing an energy-based understanding^66^ of the costs of overwintering with alternative strategies and conditions will be important for generating a complete seasonal view of circumpolar growth potential. The diet of krill can vary with size, which may affect the realised growth rates. Such variation is partially captured in the current model in which the potential growth is a function of krill size so that the relationship with food (chlorophyll *a*) changes with size. However, more detailed analyses and models of how growth rates of krill vary during development and with different food sources and conditions will be important for understanding population processes throughout the year across the whole of the Southern Ocean.

Our results indicate that seasonal-spatial and interannual variation needs to be taken into account in the next stage of the development of projections of change in Antarctic krill. In the more northern regions the majority of growth can occur before January, while further south the opposite is true. Climate-related interannual variability is a major feature of the Southern Ocean and our results indicate it will influence krill growth and hence also development. Including insights from this study of how krill growth and associated development varies under different conditions between years will be important in developing a more complete understanding of the impacts of climate variability in these ecosystems^21, 48, 67^. Including these processes is also likely to be crucial for developing ecologically useful projections of the impacts of future climate-driven change. Based on our model analyses, under warming scenarios, krill would be expected to be smaller in northern regions in the future (cf. Figs 4 and S4), and the main areas of positive potential growth will occur further south, although this will also depend on changes in primary production and food availability (see also^24^). However, we also note that physical constraints on seasonality in irradiance mean that southern areas will not sustain the extended seasonal growth currently possible in the most northern areas. This would further reduce annual growth rates resulting in a population size structure dominated by smaller krill, reducing the reproductive potential of female krill (Figure S2) and the overall secondary production associated with krill. Such changes would affect regional availability, especially in northern areas such as around South Georgia in the Scotia Sea, that are currently crucial for breeding seabirds and marine mammals, potentially making them a less suitable habitat in the future.

This paper presents a new perspective on the distribution of krill, demonstrating that regional differences in seasonal growth dynamics constrain where krill can successfully grow and reproduce in the Southern Ocean. Our findings provide a first quantitative basis for why not all areas can support the growth and maintenance of the very largest krill and for making predictions of size structure. This has fundamental implications about the capacity of different regional ecosystems to maintain secondary production and support large-bodied predators. Our results demonstrate that it is crucial that seasonal variation is included in models for development of projections of the impacts of physical change in the Southern Ocean in order to reveal shifts in the spatial structure and functioning of ecosystems.

## Methods and Data

### Empirical model of krill growth

We use the empirically derived model of krill growth (Atkinson *et al*. 2006; Model 3^37^; see their Table 5) to estimate the potential growth (change in size, mm) across the circumpolar Southern Ocean by month for different sized krill^11^. Our focus is on growth potential and the size of the krill rather than overall gross production potential (c.f. ref. 24). The growth rate (*ΔL* mm day^−1^) is given by:

1 $$\Delta L=\alpha +(\beta L)+(\gamma {L}^{2})+(\delta P/(\varepsilon +P))+(\zeta T)+(\eta {T}^{2})$$

where *L* is the krill length (mm), *P* is the concentration of chlorophyll *a* (mg m^−3^; a proxy for food availability) and *T* is the sea-surface temperature (SST) (°C). Mean parameter values are given in Table S3 along with associated standard errors.

### Circumpolar data

Our objective was to consider circumpolar scale differences in growth potential and also to examine seasonal variation. To ensure a balance between the appropriate spatial resolution and minimising the number of cells where values were missing we used 1° horizontal resolution sea surface temperature (SST) and chlorophyll a data. We obtained 1° spatially resolved mean monthly SST and sea-ice concentration data **(** http://iridl.ldeo.columbia.edu/filters/.NINO/SOURCES/.NOAA/.NCEP/.EMC/.CMB/.GLOBAL/.Reyn_SmithOIv2/ ^68^) and derived monthly climatologies for the period from November 1981 to January 2015. These are standard datasets that provide consistently-derived SST and sea-ice series. The series have been used in a range of previous analyses of interannual variability and provide a good representation of regional variation in the Southern Ocean^67^. We obtained SeaWiFS OC4v6 data at monthly 9 km resolution for the period from September 1997 to December 2010 (https://giovanni.gsfc.nasa.gov/giovanni/) and derived 1° spatially resolved data using natural neighbour interpolation (Matlab function: scatteredInterpolant). This dataset was used in the derivation of the original krill growth model and also provides a useful long term series for investigation of seasonality and interannual variability.

Estimates of the circumpolar distribution and abundance of Antarctic krill were based on historically derived net-samples^69^ (doi.org/brg8). These data provide the current best estimates of distribution and abundance of krill, although there were large variations in sampling intensity and gaps in the base data.

### Circumpolar potential growth

Equation 1 was used to estimate the growth potential for each 1° by 1° latitude-longitude grid cell for each month (mm month^−1^) for three reference sizes of krill of 20 mm, 40 mm and 60 mm. SST values <−1 °C were capped at −1 °C and the model was run for all grid cells with SST in the range −1 °C to 5 °C, the range over which the model was derived.

Growth potential was calculated in two ways. In the first we calculated growth rates for each month only for months and cells where both SST and chlorophyll *a* values were present. In the second calculation for the months and locations where SST data were available but chlorophyll data were missing, we assumed the chlorophyll *a* concentration was 0.0 mg m^−3^. Area-weighted mean growth rates were derived to generate summary estimates of circumpolar mean monthly growth rates (September to April; May to August were excluded because of extensive missing data due to cloud and sea ice cover). To calculate individual growth curves at specific localities, we undertook linear interpolation of the monthly (1° spatially resolved) values to derive estimates of daily SST and chlorophyll *a*. Equation 1 was applied iteratively to calculate daily growth rate (mm d^−1^). Calculations were initiated when SST and chlorophyll *a* values were both first available after the 1^st^ September. When there was no chlorophyll *a* estimate available later in the season a concentration of 0.0 mg chl *a* m^−3^ was assumed.

We assessed the potential importance of interannual variability in growth conditions in determining the growth and development of krill. There are lots of gaps in the monthly chlorophyll *a* concentration fields so we focused on the high-growth location of the Scotia Sea (40°W, 54°S), where monthly SeaWiFS data coverage was adequate (for the period from November to April) and examined how krill size changed in different years over a decade from 1998 to 2007. We initiated the calculations in November and calculated size change through to April because data were available for the whole period for most years (missing chlorophyll *a* estimates were replaced with a concentration of 0.0 mg chl *a* m^−3^). This start date does, however, miss the early spring months, which we have shown are likely to be important in this area (Figs 1 and 4).

To examine the impact of interannual variation in growth rate on biomass we investigated the effect of changes in the krill length-frequency distribution. Based on the analyses of interannual variation (Fig. 5) we assumed that growth rate variation was consistent across all size classes within a year and generated a size difference in a range of ±4 mm in December-January between years. We calculated the mass of all individuals in the observed length frequencies^48^ using the length to mass conversion relationship shown in Figure S2. We then recalculated the total mass assuming the length-frequency was shifted by ±4 mm. We derived mass ratios relative to the mean to scale the observed biomass estimates (see Table 2 in ref. 48) and generate a low and high growth rate biomass estimate for each year (Table S2).

Calculations presented in maps were undertaken in Matlab software version R2013a (www.mathworks.com) and the maps generated using M_Map mapping toolbox version 1.4f (https://www.eoas.ubc.ca/~rich/map.html). Calculations of growth curves (Figs 4 and 5) were undertaken in Wolfram Mathematica version 11 (www.wolfram.com/mathematica).

## Electronic supplementary material

Supplementary Information
