# Supplementary material for: Restricted regions of enhanced growth of Antarctic krill in the circumpolar Southern Ocean
Source: Sci Rep. 2017 Jul 31;7:6963. doi: 10.1038/s41598-017-07205-9 (PMC5537218; doi:10.1038/s41598-017-07205-9)
Supplement: Supplementary file 1 — Supplementary Information [file 41598_2017_7205_MOESM1_ESM.pdf]

Restricted regions of enhanced growth of Antarctic krill in the circumpolar Southern Ocean

E J Murphy, S E Thorpe, G A Tarling, J L Watkins, S Fielding and P Underwood

Supplementary information

Seasonal variation: Table S1 shows the mean potential growth rate (mm month^{-1}) of different sized krill during different seasons (Std dev = standard deviation; Range = maximum – minimum growth rate; N = number of grid cells). Calculations are shown for Sep-Nov, Dec-Feb, Mar-Apr and for the overall period from Aug-Apr. Potential growth rates were calculated over the Southern Ocean in areas where both temperature and chlorophyll *a* data are available (as in Figure 1). Area (km^2) is shown for each calculation and is also given as the proportion of the summer (Dec-Feb) and total annual (Aug to Apr) areas. Estimated rates are given for calculations over all cells, cells where growth is positive and where growth is >2 mm month$^{-1.}$

Interannual variation: Figure S1 shows the climatological mean sea surface temperature (SST) and chlorophyll a concentration (±1 standard deviation) for the period from 1997/1998 to 2006/2007 at 54°S, 40°W, with the anomaly plots for each year. These data were used to derive the interannual growth curves shown in main paper Figure 5. Scaled estimates of biomass for each year[1] for low and high growth rate conditions are given in Table S2.

Mass and egg number length conversions: Figure S2 shows the change in relative mass and fecundity (egg number) of a krill of length L compared to a krill of length 40 mm. We used the length (L) to wet mass (m_w) conversion of Morris et al., 1988[2] ($m_w = 3.85 \times 10^{-6} L^{3.20}$) and the length to egg number conversion of Tarling et al. 2007[3] (Egg No. = $10^{(4.55 \log10 L - 3.88)}$).

Sensitivity of the empirical model: Optimum growth rates vary with animal size, but occur generally between temperatures -1 to 2 ^{o}C and chlorophyll *a* concentrations > 1.0 mg m^{-3} (Figure S3). For chlorophyll *a* concentrations >1.0 mg m^{-3} there is little change in the potential rate of growth, but the model is particularly sensitive to the change in chlorophyll *a* concentration from < 0.1 mg m^{-3} to > 1 mg chl *a* m^{-3} (Figure S3[4]). Above ~1°C growth rates decline and this is particularly rapid above ~2°C and also below ~-1°C. Calculations using parameter values of plus and minus one standard error demonstrate the wide variation in

estimated growth rates from which the model was derived (Figure S3 and Table S4[4]). However, although absolute rates change with different parameterisations, the general patterns of sensitivity to changes in SST and chlorophyll concentration are similar (Figure S3), which supports the use of the model for analyses of variations in growth rates associated with their size and with regional SST and food concentrations.

Warming scenario: Figure S4 shows growth trajectories for two sites (as shown in main paper Figure 4) under scenario of a 1°C increase in SST.

Calculations presented in Table S1 were undertaken in Matlab software version R2013a (www.mathworks.com). Calculations presented in Tables S2 and S4 and in Supplementary Figures S1 to S4 were undertaken in Wolfram Mathematica version 11 (www.wolfram.com/mathematica). Supplementary Figures S1 to S4 were created using Wolfram Mathematica version 11 (www.wolfram.com/mathematica)

Supplementary figure captions

Figure S1. Seasonal SST and chlorophyll *a* concentrations for the period from 1998/1999 to 2007/2008 (mean and standard deviation). Anomalies about the climatological monthly means are shown for each year.

Figure S2. Change in relative mass (black line) and fecundity (egg number, blue line) of a krill of length *L* compared to a krill of length 40 mm.

Figure S3. Sensitivity plots of the growth rate (mm d^{-1}) of krill for 20 mm (top panels), 40 mm (middle panels) and 60 mm animals (bottom panels). Plots are shown for mean parameterisations (middle), mean parameterisations minus one standard error (left) and mean parameterisations plus one standard error (right) of the growth equation (Main paper equation 1[4] and Table S3).

Figure S4. As Figure 4, assuming a circumpolar warming in SST of 1 ^{o}C applied to all grid cells. Model estimated seasonal growth curves (September to mid-April) at different latitudes along two longitudes: 60°E (Indian Ocean sector, left panels) and 40°W

(Scotia/Weddell Seas, right panels). Growth curves are shown for krill with initial sizes of 20 mm (upper panels), 40 mm (centre panels) and 60 mm (bottom panels). The colours show the growth curves for different latitudes (see lower panels for key).

References

1. Fielding S, *et al.* Interannual variability in Antarctic krill (Euphausia superba) density at South Georgia, Southern Ocean: 1997-2013. *ICES J. Mar. Sci.* **71**, 2578-2588 (2014).
2. Morris DJ, Watkins JL, Ricketts C, Buchholz F, Priddle J. An assessment of the merits of length and weight measurements of Antarctic krill *Euphausia superba*. *British Antarctic Survey Bull.*, 27-50 (1988).
3. Tarling GA, Cuzin-Roudy J, Thorpe SE, Shreeve RS, Ward P, Murphy EJ. Recruitment of Antarctic krill *Euphausia superba* in the South Georgia region: adult fecundity and the fate of larvae. *Mar. Ecol.-Prog. Ser.* **331**, 161-179 (2007).
4. Atkinson A, *et al.* Natural growth rates in Antarctic krill (Euphausia superba): II. Predictive models based on food, temperature, body length, sex, and maturity stage. *Limnol. Oceanog.* **51**, 973-987 (2006).

Months	Potential growth rates (mm month^{-1})	Body length 20 mm All cells	20 mm Positive growth	20 mm Growth > 2	40 mm All cells	40 mm Positive growth	40 mm Growth > 2	60 mm All cells	60 mm Positive growth	60 mm Growth > 2
August-April	Mean	1.93	2.77	3.73	0.92	2.09	3.72	-1.56	1.69	3.40
	Std dev	2.45	1.79	1.61	2.45	1.68	1.51	2.45	1.44	1.07
	Range	16.16	10.11	8.11	16.16	9.08	7.08	16.16	6.54	4.54
	N	40405	33757	21737	40405	29923	12852	40405	10219	3839
	Area (x 10^7 km^2)	24.98	20.29	12.59	24.98	17.74	7.02	24.98	5.42	1.82
Sep-Nov (Spring)	Mean	1.73	2.47	3.18	0.72	1.69	3.17	-1.77	1.19	3.00
	Std dev	2.06	1.33	1.14	2.06	1.21	1.14	2.07	1.11	0.82
	Range	14.61	9.56	7.56	14.61	8.57	6.57	14.61	6.11	4.10
	N	11798	9909	6208	11798	8880	2656	11798	1763	396
	Area (x 10^7 km^2)	7.78	6.40	4.00	7.78	5.71	1.68	7.78	1.09	0.23
	Proportion summer area	0.82	0.79	0.69	0.82	0.79	0.40	0.82	0.30	0.16
	Proportion Aug to Apr area	0.31	0.32	0.32	0.31	0.32	0.24	0.31	0.20	0.13
Dec-Feb (Summer)	Mean	2.72	3.46	4.38	1.72	2.80	4.06	-0.74	1.93	3.50
	Std dev	2.68	2.12	1.80	2.67	1.98	1.62	2.66	1.52	1.10
	Range	14.48	10.11	8.11	14.41	9.08	7.08	14.41	6.54	4.54
	N	16621	14512	10831	16621	13221	8220	16621	7091	3126
	Area (x 10^7 km^2)	9.53	8.10	5.80	9.53	7.26	4.25	9.53	3.60	1.44
	Proportion summer area	1.00	1.00	1.00	1.00	1.00	1.00	1.00	1.00	1.00
	Proportion Aug to Apr area	0.38	0.40	0.46	0.38	0.41	0.61	0.38	0.66	0.79
Mar-Apr (Autumn)	Mean	1.31	2.28	3.34	0.29	1.62	3.29	-2.22	1.26	3.14
	Std dev	2.25	1.45	1.26	2.24	1.33	1.21	2.24	1.17	0.94
	Range	14.77	9.48	7.48	14.73	8.45	6.45	14.66	5.91	3.90
	N	9354	7411	3908	9354	6242	1927	9354	1348	317
	Area (x 10^7 km^2)	5.76	4.41	2.23	5.76	3.65	1.06	5.76	0.72	0.15
	Proportion summer area	0.60	0.54	0.38	0.60	0.50	0.25	0.60	0.20	0.10
	Proportion Aug to Apr area	0.23	0.22	0.18	0.23	0.21	0.15	0.23	0.13	0.08

Table S1. Seasonal changes in the mean potential growth rate (mm month^{-1}) of different sized krill (Std dev = standard deviation; Range = maximum – minimum growth rate; N = number of grid cells). Calculated over the Southern Ocean (as in Figure 1) in areas where both temperature and chlorophyll *a* data are available (Area (km^2) is shown for each calculation, and as a proportion of the summer (Dec-Feb) and total annual (Aug to Apr) areas. Calculations are shown over all cells, cells where growth is positive and where growth is > 2 mm month^{-1}.

year	low growth	mean biomass (g m^{-2})	high growth
1997	23.9	31.7	41.0
1998	29.1	38.8	50.7
1999	7.6	9.7	12.2
2000	2.0	2.7	3.6
2001	27.5	36.7	48.0
2002	100.8	137.0	181.5
2003	63.0	84.6	111.0
2004	19.9	26.1	33.7
2005	69.6	89.4	112.9
2006	89.5	119.1	155.0
2007	44.5	61.1	81.7
2009	22.3	28.8	36.5
2010	11.3	15.0	19.6
2011	44.0	59.0	77.3
2012	68.8	90.1	115.6

Table S2. Estimated mean biomass of krill (g m^{-2}) around South Georgia from Fielding *et al.*[1] with scaled biomass estimates assuming low and high growth rates (±4mm).

Parameter	mean	s.e.
α	-0.066	0.165
β	0.002	0.0066
γ	-0.000061	-0.000077
δ	0.385	0.091
ε	0.328	0.246
ζ	0.0078	0.017
η	-0.0101	-0.00045

Table S3. The estimated mean and standard errors of the parameter values of the growth equation (Main paper equation 1).

Krill size (mm)	Growth rate (mm d^{-1})		
	-1 s.e.	Mean parameters	+1 s.e.
20	-0.05	0.24	0.53
40	-0.12	0.21	0.53
60	-0.18	0.13	0.43

Table S4. Estimated growth rate of three different sized krill (20 mm, 40 mm and 60 mm) for the mean parameterisation and for ± 1 s.e (Table S3). Values were estimated at SST of 0.5 ^{o}C and a chlorophyll concentration of 0.5 mg m^{-3}.

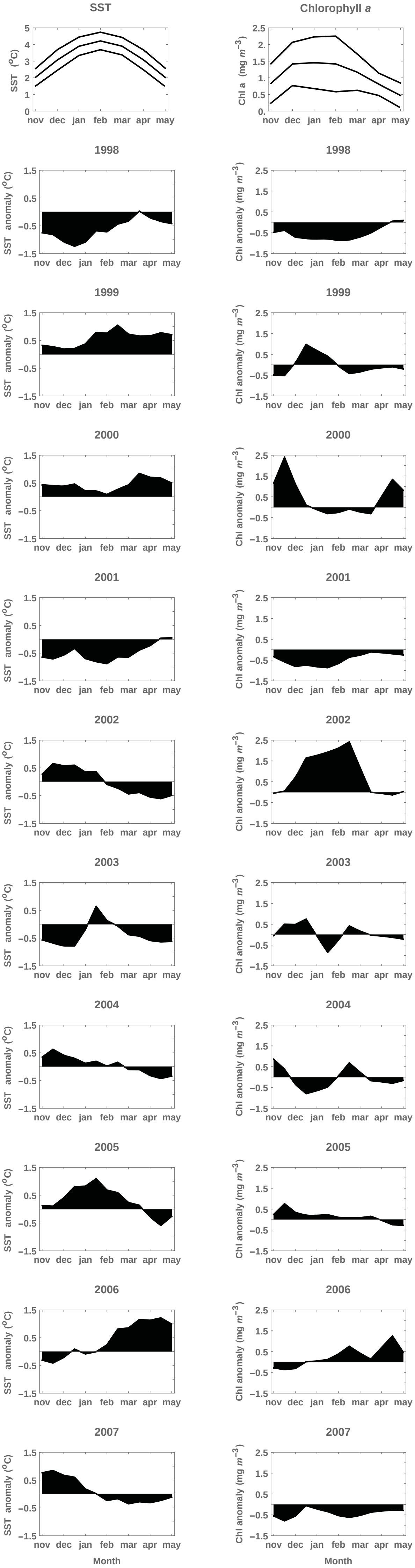
SST
Chlorophyll a
1998
1999
2000
2001
2002
2003
2004
2005
2006
2007
SST (°C)
Chl a (mg m⁻³)
SST anomaly (°C)
Chl anomaly (mg m⁻³)
nov dec jan feb mar apr may
Month

Figure S1

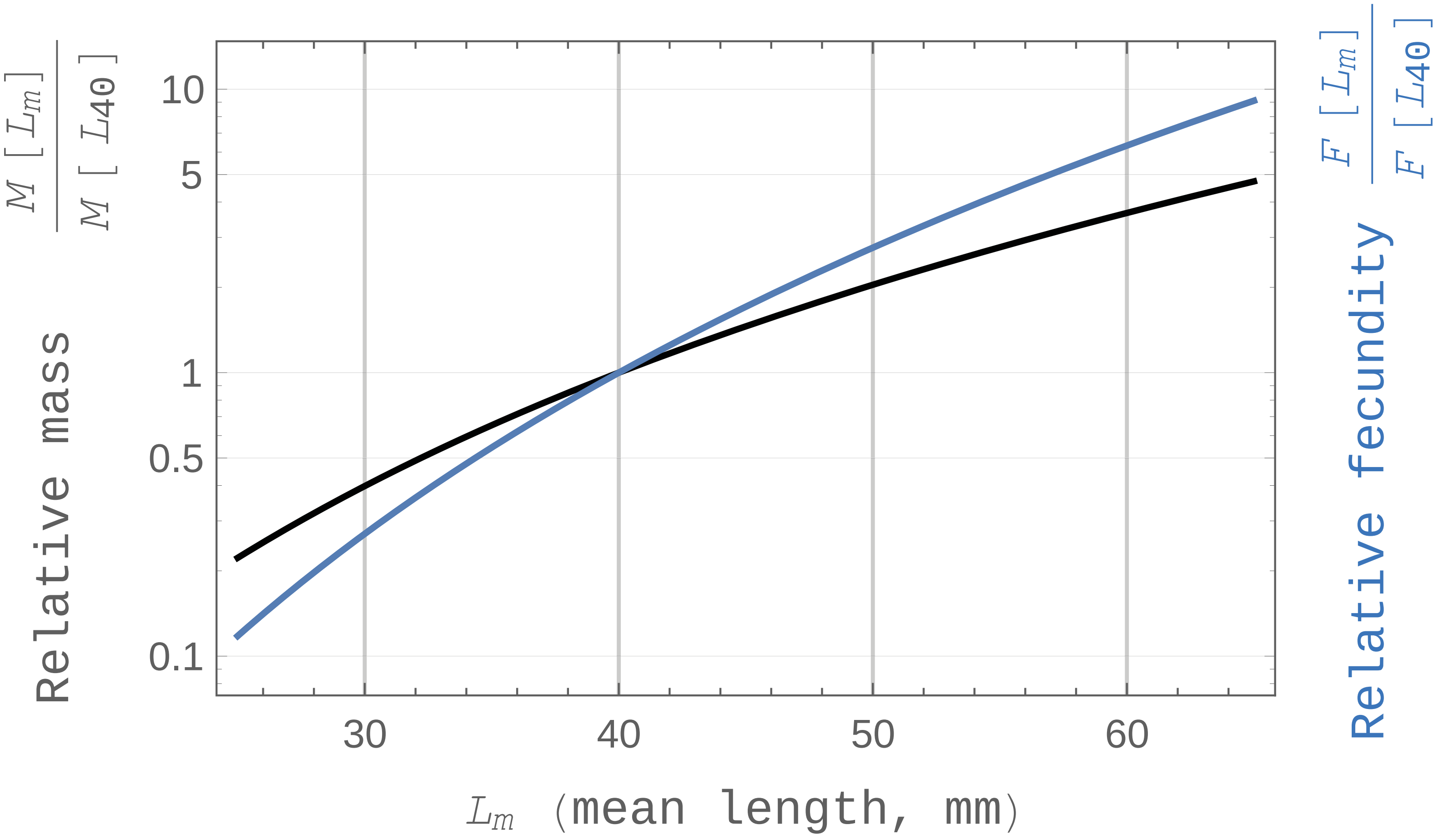

Relative mass
$\frac{M[L_m]}{M[L_{40}]}$
10
5
1
0.5
0.1
30
40
50
60
L_m (mean length, mm)
Relative fecundity
$\frac{F[L_m]}{F[L_{40}]}$

Figure S2

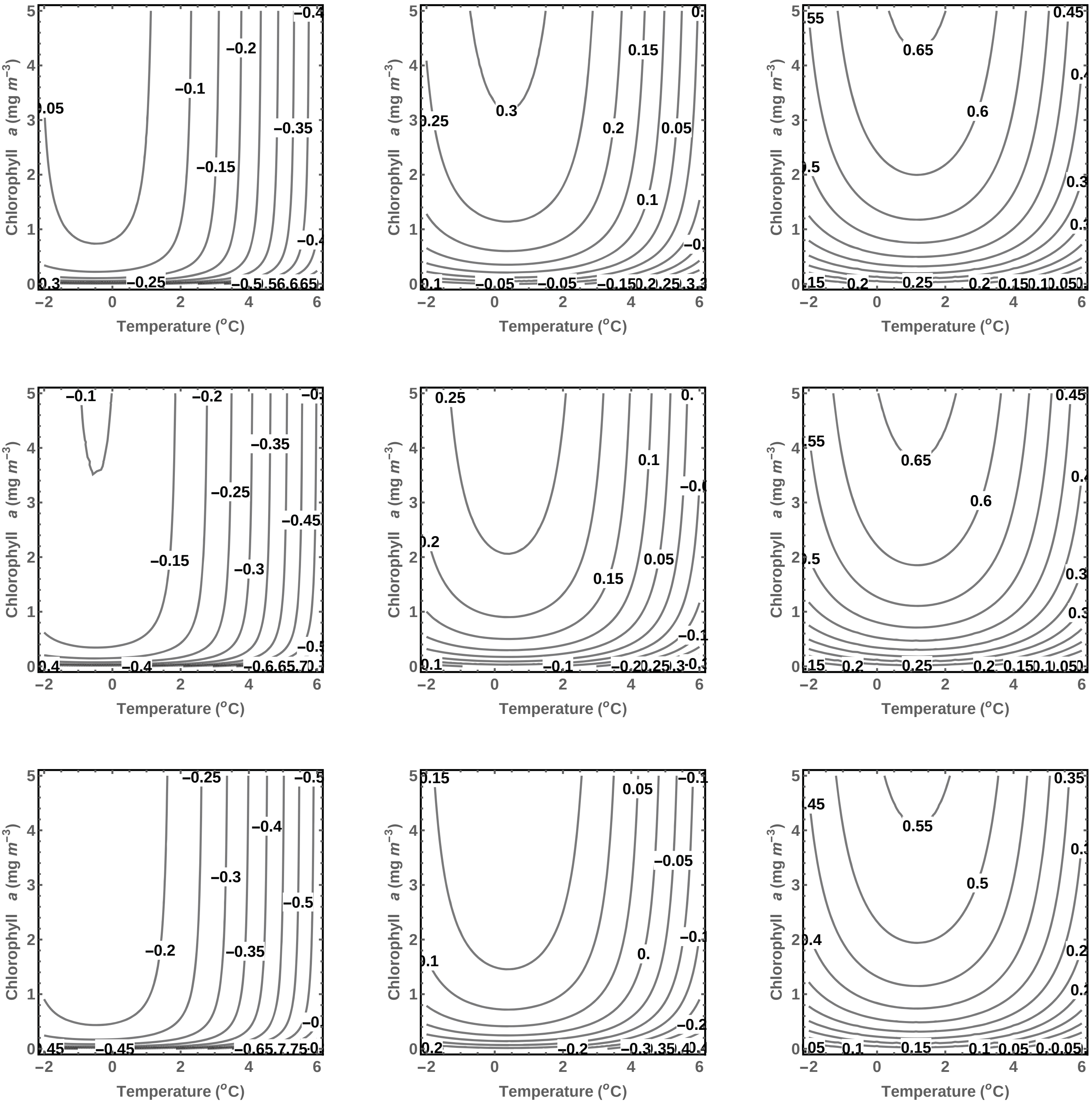

Figure S3

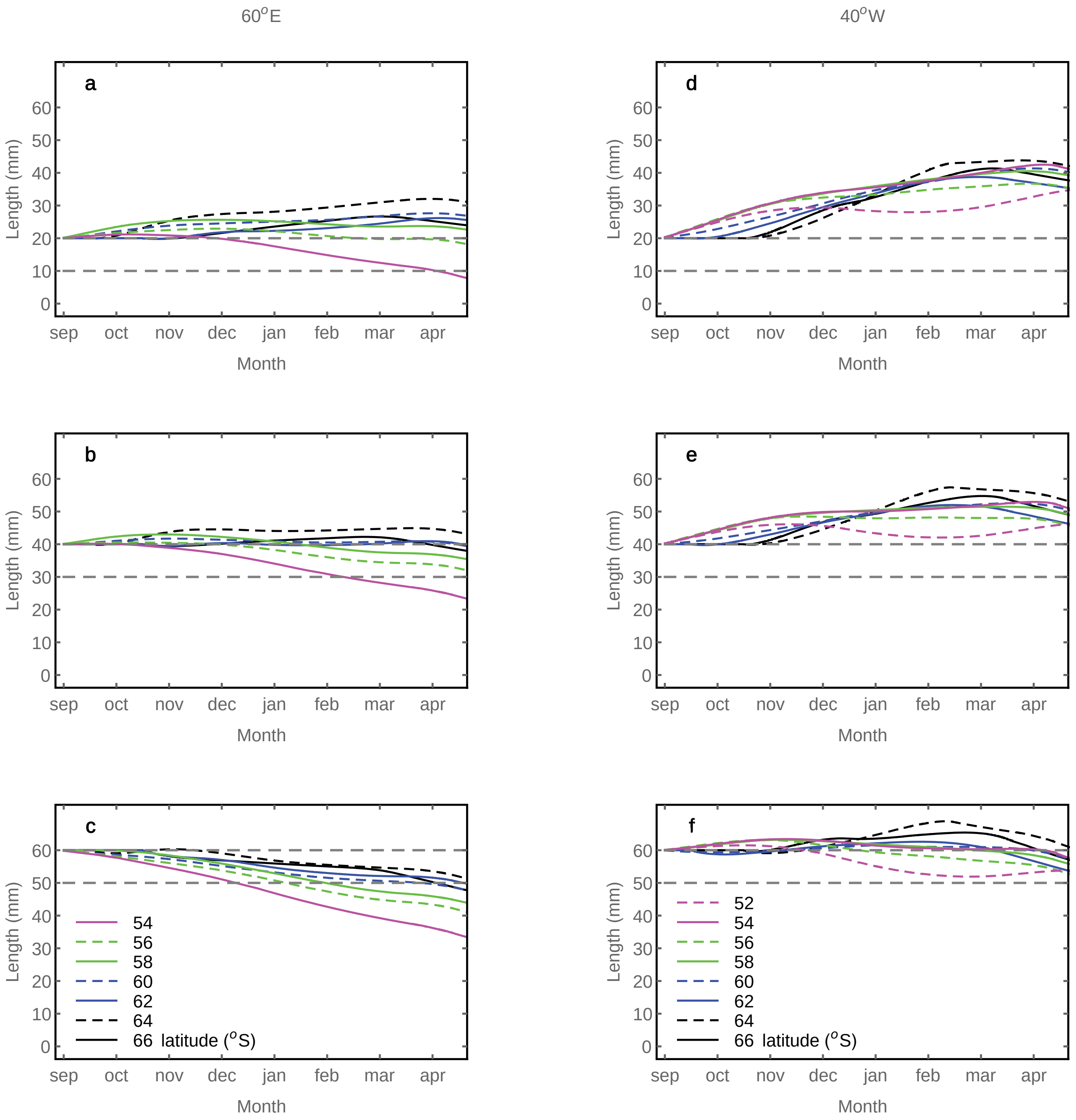
60°E
40°W
a
b
c
d
e
f
Length (mm)
Month
sep
oct
nov
dec
jan
feb
mar
apr
0
10
20
30
40
50
60
52
54
56
58
60
62
64
66 latitude (°S)

Figure S4
